# Activation of TRPV1-Expressing Renal Sensory Nerves of Rats with N-Oleoyldopamine Attenuates High-Fat-Diet-Induced Impairment of Renal Function

**DOI:** 10.3390/ijms24076207

**Published:** 2023-03-25

**Authors:** Shuang-Quan Yu, Shuangtao Ma, Donna H. Wang

**Affiliations:** 1 Division of Nanomedicine and Molecular Intervention, Department of Medicine, Michigan State University, East Lansing, MI 48824, USA; shuangquanyu@yahoo.com (S.-Q.Y.); mashuang@msu.edu (S.M.); 2 Neuroscience Program, Michigan State University, East Lansing, MI 48824, USA; 3 Cell & Molecular Biology Program, Michigan State University, East Lansing, MI 48824, USA

**Keywords:** N-oleoyldopamine, TRPV1, high-fat diet, obesity, hypertension

## Abstract

Enhanced renal sympathetic nerve activity (RSNA) contributes to obesity-induced renal disease, while the role of afferent renal nerve activity (ARNA) is not fully understood. The present study tested the hypothesis that activating the transient receptor potential vanilloid 1 (TRPV1) channel in afferent renal nerves suppresses RSNA and prevents renal dysfunction and hypertension in obese rats. N-oleoyldopamine (OLDA, 1 ng/kg, daily) was administrated intrathecally (T8-L3) via an indwelled catheter to chronically activate, TRPV1-positive afferent renal nerves in rats fed a chow diet or high-fat diet (HFD) for 8 weeks. HFD intake significantly increased the body weight, impaired glucose and insulin tolerance, decreased creatinine clearance, and elevated systolic blood pressure in rats compared with the levels of the chow-fed rats (all *p* < 0.05). An intrathecal OLDA treatment for 8 weeks did not affect the fasting glucose level, glucose tolerance, and insulin tolerance in rats fed either chow or HFD. As expected, the chronic OLDA treatment significantly increased the levels of plasma calcitonin gene-related peptide and substance P and ARNA in the HFD-fed rats (all *p* < 0.05). Interestingly, the OLDA treatment decreased the urinary norepinephrine level and RSNA in rats fed HFD (both *p* < 0.05). Importantly, the OLDA treatment attenuated HFD-induced decreases in creatinine clearance and urinary Na^+^ excretion and increases in the plasma urea level, urinary albumin level, and systolic blood pressure at the end of an 8-week treatment (all *p* < 0.05). Taken together, the intrathecal administration of OLDA ameliorates the enhancement of RSNA, renal dysfunction, and hypertension in obese rats. These findings shed light on the roles of TRPV1-positive renal afferent nerves in obesity-related renal dysfunction and hypertension.

## 1. Introduction

Obesity is a major risk factor for hypertension and chronic renal disease [1]. Enhanced renal inflammation, oxidative stress, and tissue injury have been observed in obese humans and animals [2]. Enhanced renal sympathetic nerve activity (RSNA) occurs during obesity, which may contribute to renal damage [2]. It has been shown that systemic sympathetic inhibition or renal denervation eliminates obesity-associated renal abnormalities or hypertension [3]. In contrast to well-studied RSNA, the role of afferent renal nerve activity (ARNA) in obesity-mediated effects is largely unknown. 

The transient receptor potential vanilloid 1 (TRPV1) channel is abundantly expressed in afferent renal nerves [4]. It has been shown that systemically activating TRPV1 lowers the blood pressure in spontaneously hypertensive rat [5]. However, the role of TRPV1-positive afferent renal nerves in obesity-induced renal injury has not been fully delineated. Our previous studies demonstrated that knockout of TRPV1 exacerbated an obesity-induced renal injury and nocturnal hypertension in mice [6,7,8]. Moreover, we found that the selective ablation of TRPV1-positive afferent renal nerves by the intrathecal injection of resiniferatoxin in rats increases the renal sympathoexcitatory responses and salt sensitivity [9,10]. In addition, our previous study showed that TRPV1-positive afferent renal nerves had a suppressing effect on renal sympathetic nerve activity [11,12]. Taken together, these findings suggest that TRPV1-positive afferent renal nerves may have a protective role against obesity-induced renal injury and hypertension. 

To explore the role of TRPV1-positive afferent renal nerves in obesity-related renal injury, we chronically and specifically activate TRPV1 channels in afferent renal nerves with an intrathecal N-oleoyldopamine (OLDA, an endogenous TRPV1 agonist) injection in the present study. OLDA was administered intrathecally to the subarachnoid space in the T8-L3 segments of the spinal cord of rats because T8-L3 segments of the spinal cord reportedly receive the most projections from dorsal root ganglia neurons, innervating the kidney [13]. The method of the intrathecal administration of drugs was applied in our other studies and showed high efficiency and selectivity of the delivery of drugs into the central terminals of dorsal root ganglia neurons, innervating the kidney [9,14,15]. 

In the present study, we tested the hypotheses that the chronically intrathecal administration of OLDA can ameliorate the high-fat-diet-(HFD)-induced enhancement of renal sympathetic nerve activity, renal dysfunction, and blood pressure elevation in rats. 

## 2. Results

### 2.1. OLDA Does Not Affect Glucose Tolerance and Insulin Sensitivity in HFD-Fed Mice

**Figure 1:**
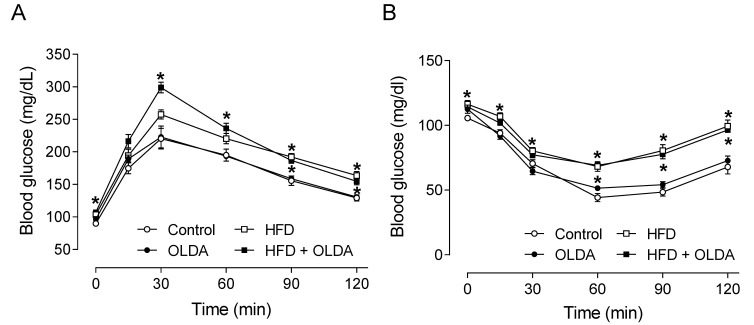
Effects of OLDA on glucose and insulin tolerance. The glucose tolerance test (**A**) and insulin tolerance test (**B**) in rats fed chow diet or high-fat diet (HFD) by the end of the 8 week treatment with or without intrathecal N-oleoyldopamine (OLDA) treatment (1 ng/kg, daily). Blood from the tail was collected to measure the level of blood glucose before and after intraperitoneal injection of glucose (2 g/kg) in rats with 15 h fasting or insulin (0.75 IU/kg) in rats with 6 h fasting, respectively. Values are means ± SE (n = 8). * *p* < 0.05 compared with that of the chow group.

As expected, HFD intake significantly increased the body weight of rats (Chow: 455 ± 20 g vs. HFD: 554 ± 20 g, *p* < 0.05), while intrathecal the OLDA treatment did not affect the body weight of either chow-fed rats (Chow: 455 ± 20 g vs. Chow + OLDA: 448 ± 11 g, *p* > 0.05) or HFD-fed rats (HFD: 554 ± 20 g vs. HFD + OLDA: 521 ± 14 g, *p* > 0.05). Similarly, OLDA did not affect the HFD-induced glucose tolerance, expressed as elevated fasting plasma blood glucose levels (Chow: 93 ± 2 mg/dL, Chow + OLDA: 91 ± 2 mg/dL, HFD: 106 ± 1 mg/dL, HFD + OLDA: 110 ± 2 mg/dL, *p* < 0.05), and 2 h post-injection blood glucose levels (Chow: 129 ± 5 mg/dL, Chow + OLDA: 131 ± 4 mg/dL, HFD: 164 ± 6 mg/dL, HFD + OLDA: 155 ± 5 mg/dL, *p* < 0.05) (Figure 1A). Similar results were obtained in the insulin tolerance test, expressed as blood glucose levels at 2h after the insulin injection (Chow: 68 ± 5 mg/dL, Chow + OLDA: 73 ± 4 mg/dL, HFD: 99 ± 5 mg/dL, HFD + OLDA: 97 ± 2 mg/dL, *p* < 0.05) (Figure 1B). 

### 2.2. OLDA Attenuates HFD-Induced Impairment in Afferent Renal Nerve Activity

**Figure 2:**
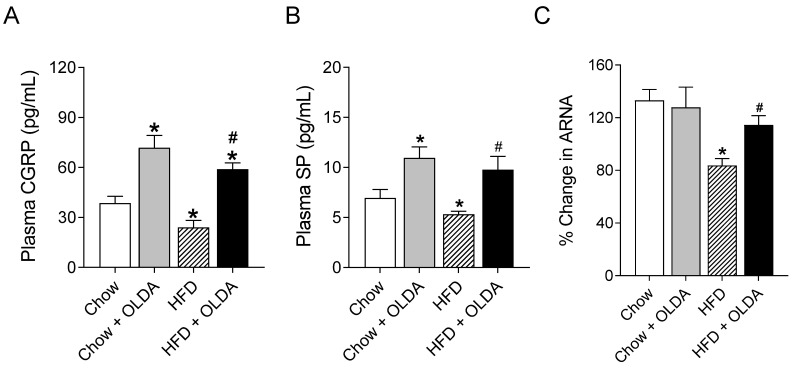
Effects of OLDA on afferent renal nerve function. The levels of plasma calcitonin gene-related peptide (CGRP) (**A**), substance P (SP) (**B**), and afferent renal nerve activity (ARNA) (**C**) response to intra-pelvis administration of capsaicin in chow-fed or high-fat-diet (HFD)-fed rats after 8 week of treatment with or without intrathecal N-oleoyldopamine (OLDA) treatment (1 ng/kg, daily). Values are means ± SE (n = 8). * *p* < 0.05 compared with that of the chow group; # *p* < 0.05 compared with that of the HFD group.

HFD significantly decreased the plasma CGRP levels in rats (*p* < 0.05), while the intrathecal treatment of OLDA for 8 weeks increased the levels of plasma CGRP in both chow-fed and HFD-fed rats (both *p* < 0.05, Figure 2A). The results of plasma SP measurements were consistent with the results of plasma CGRP (Figure 2B). HFD significantly suppressed afferent renal nerve activity in response to the intra-pelvis perfusion of capsaicin, a TRPV1 agonist (*p* < 0.05), which was abolished by the OLDA treatment (*p* < 0.05). 

### 2.3. OLDA Blunts HFD-Induced Enhancement of Renal Sympathetic Nerve Activity

**Figure 3:**
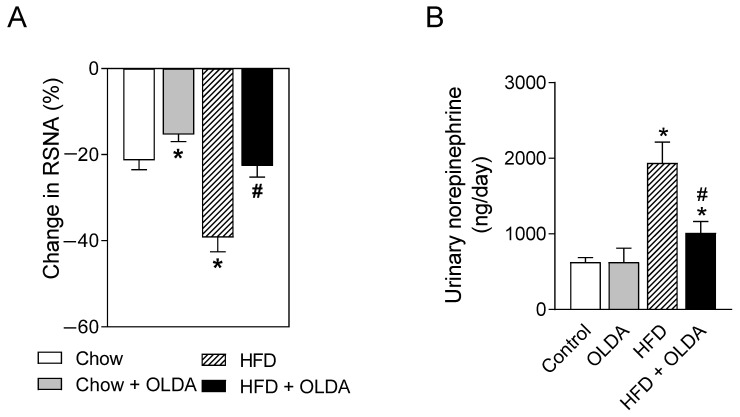
Effects of OLDA on renal sympathetic nerve activity. The renal sympathetic nerve activity (RSNA) response to intrathecal administration of muscimol (**A**) and the level of urinary norepinephrine (**B**) in chow-fed or high-fat-diet (HFD)-fed rats after 8 week of treatment with or without intrathecal N-oleoyldopamine (OLDA) treatment (1 ng/kg, daily). Values are means ± SE (n = 8). * *p* < 0.05 compared with that of the chow group; # *p* < 0.05 compared with that of the HFD group.

HFD remarkably increased the renal sympathetic nerve activity reflected by the response to the intrathecal administration of muscimol (3 nmol/kg, a γ-Aminobutyric acid sub-type A receptor agonist) (*p* < 0.05), which was reversed by the OLDA treatment (*p* < 0.05) (Figure 3A). Consistently, the OLDA treatment attenuated HFD intake-induced increases in the levels of urinary norepinephrine (Figure 3B).

### 2.4. OLDA Attenuates HFD-Induced Renal Dysfunction 

**Figure 4:**
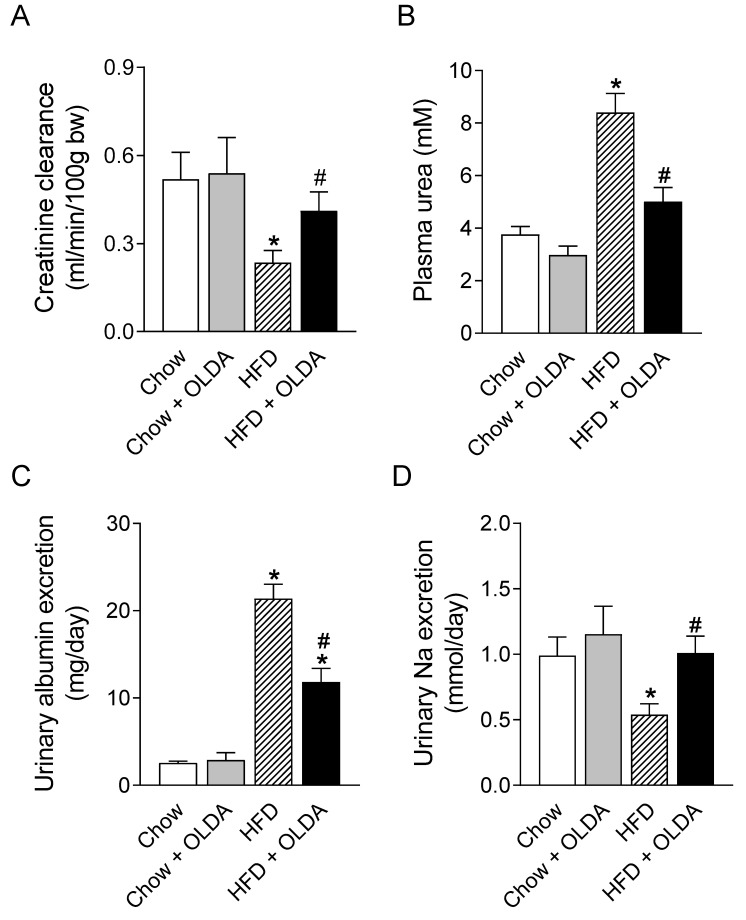
Effects of OLDA on renal function. Creatinine clearance (**A**), plasma urea level (**B**), urinary albumin excretion (**C**), and urinary sodium excretion (**D**) in chow-fed or high-fat-diet (HFD)-fed rats after 8 week of treatment with or without intrathecal N-oleoyldopamine (OLDA) treatment (1 ng/kg, daily). Values are means ± SE (n = 8). * *p* < 0.05 compared with that of the chow group; # *p* < 0.05 compared with that of the HFD group.

HFD significantly decreased the rate of creatinine clearance and urinary sodium excretion and increased the plasma urea level and urinary albumin excretion in rats (Figure 4A–D). The OLDA treatment prevented or attenuated HFD intake-induced decreases in creatinine clearance (Figure 4A, *p* < 0.05) and urinary Na^+^ excretion (Figure 4D, *p* < 0.05) and increases in the levels of plasma urea (Figure 4B, *p* < 0.05) and urinary albumin (Figure 4C, *p* < 0.05). 

### 2.5. OLDA Prevents HFD-Induced Development of Hypertension

**Figure 5:**
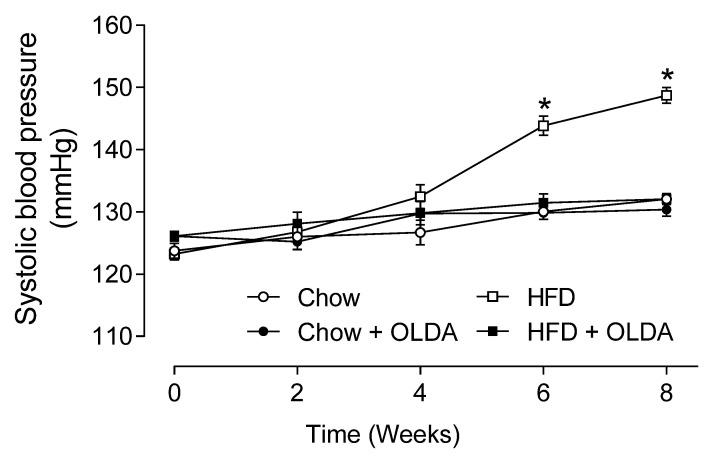
Effects of OLDA on systolic blood pressure. Tail-cuff systolic blood pressure in chow-fed or high-fat-diet (HFD)-fed rats during 8 week of treatment with or without intrathecal N-oleoyldopamine (OLDA) treatment (1 ng/kg, daily). Values are means ± SE (n = 8). * *p* < 0.05 compared with that of the chow group.

Rats fed HFD developed hypertension in the 6th and 8th weeks of the experiment, while hypertension was prevented by the OLDA treatment (systolic blood pressure in the 8th week, Chow: 132 ± 1, Chow + OLDA: 130 ± 1, HFD: 149 ± 1, HFD+OLDA: 132 ± 1 mmHg, *p* < 0.05) (Figure 5). 

## 3. Discussion

The present study found that intrathecal treatment with OLDA prevented HFD intake-induced decreases in ARNA and increases in RSNA. Importantly, the chronical OLDA treatment attenuated HFD intake-induced renal injuries and the development of hypertension. 

Obesity-induced sympathetic overactivity is well documented; however, its mechanism is not fully understood. We observed that TRPV1-mediated ARNA and the levels of plasma CGRP and SP, markers for the activity of TRPV1-positive afferent nerves, were decreased by HFD intake. These data indicate that TRPV1-positive afferent renal nerves are impaired in obesity. Meanwhile, HFD intake enhanced the RSNA and urinary norepinephrine levels, indicating sympathetic overactivity. Moreover, the activation of ARNA with the intrathecal OLDA treatment almost abolished the HFD-induced enhancement of RSNA. These findings suggest that obesity-related sympathetic overactivity may be due to the impairment of ARNA. Since, RSNA enhancement is, at least partially, responsible for renal injury, renal dysfunction, and hypertension in obesity; activating ARNA may reverse the renal complications in obesity. This is consistent with our previous studies in rats with high-salt diet intake-induced renal sympathetic overactivity [11,16,17,18,19]. 

The present study found that the OLDA treatment prevented HFD-induced decreases in TRPV1-mediated ARNA and the levels of plasma CGRP and SP, suggesting that the TRPV1-positive afferent renal nerves were protected from HFD by the intrathecal treatment with OLDA. In addition, OLDA also prevented the HFD-induced enhancement of RSNA and increased urinary norepinephrine levels and abolished HFD-induced renal functional impairment and hypertension. Taken together, the OLDA treatment prevented the occurrence of renal injury and hypertension in HFD-fed rats probably via protecting the TRPV1-positive afferent renal nerves first, and then recovering the suppressing effects of ARNA on RSNA. 

It has been reported that activation of TRPV1 may protect tissue injury by increasing the amount of CGRP that is released [20,21,22]. CGRP is released from TRPV1-positive afferent renal nerves in this study via two pathways. First, OLDA, as a lipid, was up-taken by the central terminals of dorsal root ganglion neurons, innervating the kidney, transported to the peripheral terminals, and then activated the TRPV1 channels in the nerve terminals, resulting in CGRP release in the kidney. Second, an action potential was produced by the activation of TRPV1 in the central terminals of DRG neurons, innervating the kidney, and transmitted to the peripheral terminals via the mechanism of the backpropagation of action potentials [23], resulting in CGRP release in the kidney. The OLDA-induced sustained release of CGRP in the kidney antagonized the HFD-induced impairment in TRPV1-positive afferent renal nerves, possibly via improving the renal blood flow nourishing afferent renal nerves. However, this mechanism needs to be clarified in future studies. 

Previous studies demonstrated that systemic OLDA treatment enhances the glucose metabolism [24]. The chronic and systemic activation of TRPV1 by dietary capsaicin lowered the fasting glucose level and reduced the impairment of glucose tolerance in obese mice by suppressing inflammatory responses and enhancing fatty acid oxidation in adipose tissue or by stimulating glucagon-like peptide-1 secretion in the intestinal cells and tissues [25,26]. However, in the present study, the intrathecal OLDA treatment did not affect HFD-induced elevations in the fasting basal blood glucose level and the impairment of glucose and insulin tolerance. This disparity suggests that OLDA’s effects on glucose metabolism are independent of the TRPV1-positive afferent renal nerves. These findings also suggested that OLDA’s effect in the present study is not mediated via its systemic regulating effects on glucose and insulin metabolism, but is most likely mediated by targeting the neurons innervating the kidney. 

In conclusion, HFD intake suppressed ARNA, increased RSNA, and induced renal dysfunction and the development of hypertension, which were prevented or attenuated by the chronic intrathecal administration of OLDA. The inhibitory effect of TRPV1-mediated ARNA on RSNA is likely weakened by HFD intake, contributing to obesity-related renal injury and hypertension. OLDA, given intrathecally to segments supplying the kidney, prevented HFD-induced impairment in TRPV1-positive afferent renal nerves, resulting in the restoration of the suppressive effects of ARNA on RSNA and the alleviation of renal dysfunction and hypertension. The present study provides evidence that the chronic activation of TRPV1-positive afferent renal nerves by OLDA has a neuroprotective effect on HFD-induced impairment in TRPV1-positive afferent renal nerves, suggesting that TRPV1-positive afferent renal nerves might play a preventive role in the development of renal dysfunction and hypertension induced by obesity. The strategy to use OLDA to activate TRPV1 intrathecally and chronically may be applied to other fields such as the protection of afferent nerves against ischemia/reperfusion-induced nerve injury. 

## 4. Materials and Methods

### 4.1. Drugs

N-Oleoyldopamine (OLDA, O2139, Sigma-Aldrich, St. Louis, MO, USA) was dissolved in saline including 0.0006% ethanol. Glucose (G7021, Sigma-Aldrich, St. Louis, MO, USA) was diluted in saline. Insulin (NDC 0002-7510-01, Humalog insulin lispro injection, Eli Lilly and Company, Indianapolis, IN, USA) was diluted in saline. Capsaicin (M2028, Sigma-Aldrich, St. Louis, MO, USA) was dissolved in saline including 0.1 % ethanol. Muscimol (M1523, Sigma-Aldrich, St. Louis, MO, USA) was dissolved in saline. 

### 4.2. Animals

Male Wistar rats (200–225 g) were purchased from Charles River Laboratory (Wilmington, MA, USA) and were housed and kept under a cycle of 12 h light/12 h dark with free access to food and water. One week after the surgery of intrathecal catheter implantation, rats were fed a normal chow diet (Chow, 5% of total calories from fat) or a high-fat diet (HFD, 60% of total calories from fat, TD. 06414, Harlan, Madison, WI, USA) with or without OLDA treatment for eight weeks. At the end of the experiments, blood and urine samples were collected for assays. All the protocols and experiments were approved by the Institutional Animals Care and Use Committee of Michigan State University (08/17-148-00). 

### 4.3. Surgery of Intrathecal Catheter Implantation and Intrathecal Injection

Intrathecal catheters were implanted in rats according to our previous description [9,14]. Briefly, rats were anesthetized with isoflurane (2–4%). A small incision was made in the nape skin, and the atlanto-occipital membrane was exposed. A small incision was made in the membrane to allow a polyethylene-10 catheter filled with 0.9% sterile saline to be inserted into the subarachnoid space of the T8 segment of the spinal cord. The catheter was sutured in place, and the incision was closed. A short length of the catheter was left externally for the injection of OLDA. Kanamycin, penicillin, and streptomycin were injected subcutaneously for 3 days after surgery to prevent infection. One week after catheter implantation, rats received the intrathecal injection of OLDA (1 ng/kg in 10 μL, once per day) or the same volume of saline for 8 consecutive weeks. 

### 4.4. Measurement of Systolic Blood Pressure

Rats were trained to be accustomed to the measurement environment for 5 days before the measurement day to minimize the effect of stress on blood pressure. The systolic blood pressure value was the average of 9 separate measurements of conscious rats using the tail cuff method (Hatteras Instruments SC1000 Blood Pressure Analysis System, Cary, NC, USA) every other week during the 8 week treatment.

### 4.5. Glucose Tolerance Test and Insulin Tolerance Test

The glucose tolerance test and insulin tolerance test were performed at the end of 8 week treatment. Rats were fasted for 15 or 6 h; glucose (2 g/kg diluted in 0.9% saline, i.p.) or insulin (0.75 IU/kg diluted in 0.9% saline, i.p.) were administered to the conscious rats, respectively. Levels of blood glucose (from the tail) at 0, 15, 30, 60, 90, and 120 min after glucose or insulin administration were measured using a glucose meter (OneTouch Ultra Test Strips, LifeScan, Milpitas, CA, USA). 

### 4.6. Plasma and Urine Assays

Blood sample from carotid artery and urine sample were collected at the end of the 8 week treatment. Plasma CGRP (CGRP EIA Kit, 589001, Cayman Chemical, Ann Arbor, MI, USA), plasma SP (SP EIA Kit, 583751, Cayman Chemical, Ann Arbor, MI, USA), urinary Na^+^ (BQ Kits, BQ 011-EAEL, San Diego, CA, USA), urinary norepinephrine (Noradrenaline EIA Kit, 40-734-350002, GenWay Biotech, San Diego, CA, USA), plasma creatinine and urine creatinine (Creatinine assay kit, K625-100, BioVision, Mountain View, CA, USA), plasma urea (Urea assay kit, K375-100, BioVision, Mountain View, CA, USA), and urinary albumin (Rat Albumin Assay Kit, 80320, Crystal Chem, Downers Grove, IL, USA) were measured using commercially available kits. Creatinine clearance was calculated. 

### 4.7. Recording of the Activity of Afferent and Efferent Renal Nerve

Recording of the activity of renal nerve was performed as described previously [11,16,17]. Briefly, rats were anesthetized with isoflurane (beginning at 4% and maintained at 2%). The renal nerve branch was exposed, sectioned, and its proximal and distal parts were attached to stainless steel electrodes for the recording of renal sympathetic nerve activity (RSNA) and afferent renal nerve activity (ARNA), respectively. The nerve was fixed to the electrodes by applying silicone elastomer (Kwik-Cast^TM^, World Precision Instruments, Sarasota, FL, USA). The signals were amplified and filtered by a pre-amplifier (Model P511, Grass Technologies, West Warwick, RI, USA) and recorded by a recorder (Gould Instruments, Cleveland, OH, USA). Renal nerve activity was integrated over l s intervals (P3 Plus software, Boise, ID, USA) and is expressed in percentage change compared to its basal value. The background activity (when the renal nerve bundle was crushed) was subtracted from all values of renal nerve activity. For ARNA recording, the sensory nerve was activated by intra-pelvis administration of capsaicin (4 μM, 20 μL/min for 3 min) via a catheter (PE50) implanted into the pelvis. For RSNA recording, the sympathetic nerve activity was suppressed by intrathecal administration of muscimol (3 nmol/kg in 10 μL), an agonist of γ-Aminobutyric acid sub-type A receptor.

### 4.8. Statistical Analysis

All data are expressed as mean ± SE. The normality of data was confirmed by the Kolmogorov–Smirnov test using the GraphPad software (Version 4). Differences among the groups were analyzed using one-way ANOVA, followed by a Bonferroni adjustment for multiple comparisons. The differences between two groups were analyzed by using the unpaired Student’s *t*-test. The differences were considered to be statistically significant at *p* < 0.05. 

## Data Availability

Not applicable.
